# Troxerutin suppresses the stemness of osteosarcoma via the CD155/SRC/β-catenin signaling axis

**DOI:** 10.1186/s11658-025-00724-8

**Published:** 2025-04-11

**Authors:** Junkai Chen, Hongbo Li, Qinglin Jin, Xiaoguang Li, Yiwen Zhang, Jingnan Shen, Gang Huang, Junqiang Yin, Changye Zou, Xinyu Li, Xin He, Xianbiao Xie, Tiao Lin

**Affiliations:** 1 Department of Musculoskeletal Oncology, The First Affiliated Hospital of Sun Yat-sen University, Guangzhou, China; 2 Guangdong Provincial Key Laboratory of Orthopedics and Traumatology, The First Affiliated Hospital of Sun Yat-sen University, Guangzhou, China; 3 Department of Musculoskeletal Oncology, Center for Orthopaedic Surgery, The Third Affiliated Hospital of Southern Medical University, Guangzhou, China; 4 Zhongshan School of Medicine, Sun Yat-sen University, Guangzhou, China; 5 Department of Urology, The First Affiliated Hospital of Wenzhou Medical University, Wenzhou, 325000 Zhejiang China; 6 Institute of Human Virology, Department of Pathogen Biology and Biosecurity, Key Laboratory of Tropical Disease Control of Ministry of Education, Zhongshan School of Medicine, Sun Yat-sen University, Guangzhou, 510080 China

**Keywords:** CD155, Osteosarcoma, β-catenin, Stemness, Troxerutin

## Abstract

**Background:**

Osteosarcoma is the most prevalent primary malignant bone tumor affecting pediatric and adolescent individuals. However, despite the passage of three decades, there has been no notable enhancement in the overall survival rate of patients with osteosarcoma. In recent years, CD155 has been reported to exhibit abnormal amplification in a range of tumors, yet the precise underlying mechanism remains elusive. The objective of this study is to investigate the role of CD155 in osteosarcoma, and to identify drugs that specifically target this molecule, thereby offering a novel direction for the treatment of osteosarcoma.

**Methods:**

The prognosis of patients with osteosarcoma with high and low expression of CD155 was verified by immunohistochemistry. CCK-8 and colony formation assays were used to detect cell proliferation and drug resistance. Transwell experiments were used to detect cell migration and invasion. The sphere formation experiment was used to evaluate the stemness of tumor cells. Additionally, in vivo animal models were utilized to assess the functional role of CD155 in a biological context. RNA-seq and co-immunoprecipitation methods were used to search for downstream target molecules and signaling pathways of CD155. Finally, virtual screening was used to find drugs targeting CD155.

**Results:**

In this study, we have established the significant amplification of CD155 in osteosarcoma. Utilizing a comprehensive array of experimental methods, including CCK-8 assay, colony formation assay, Transwell assay, and in vivo animal models, we unequivocally demonstrate that CD155 significantly potentiates the malignancy of osteosarcoma both in vitro and in vivo. Additionally, our findings reveal that CD155 promotes osteosarcoma stemness by modulating the Wnt/β-catenin signaling pathway. Advanced molecular techniques, such as RNA sequencing and co-immunoprecipitation, have been instrumental in elucidating the mechanism of CD155 in activating the Wnt/β-catenin pathway via the SRC/AKT/GSK3β signaling axis, thereby enhancing the stem-cell-like properties of osteosarcoma cells. To explore targeted therapeutic options, we conducted virtual screening and identified troxerutin as a promising CD155 inhibitor.

**Conclusions:**

Our findings reveal that troxerutin effectively inhibits CD155, attenuates the SRC/AKT/GSK3β signaling cascade, diminishes the nuclear localization of β-catenin, and consequently mitigates osteosarcoma stemness. These discoveries position troxerutin as a promising candidate for targeted osteosarcoma therapy.

**Supplementary Information:**

The online version contains supplementary material available at 10.1186/s11658-025-00724-8.

## Highlights

- The overexpression of CD155 is linked to unfavorable prognosis in osteosarcoma.
- CD155 facilitates the stemness of osteosarcoma.
- CD155 can facilitate the nuclear translocation of β-catenin.
- Troxerutin can effectively inhibit CD155 and suppress tumor stemness.

**Supplementary Information:**

The online version contains supplementary material available at 10.1186/s11658-025-00724-8.

## Introduction

Osteosarcoma (OS) is the most frequent primary malignant bone tumor affecting children and adolescents, with a global incidence of approximately 2–3 cases per million per year [1–4]. Originating primarily from mesenchymal cells residing in the long bones adjacent to the knee joint, OS is distinguished by its significant malignancy and propensity for pulmonary metastasis [5]. The current therapeutic regimen, combining surgical resection with chemotherapy, has improved the 5-year survival rate for localized cases to 60–70%. However, the stagnation of overall survival rates over the past three decades calls for deeper exploration into the pathogenesis of OS to foster novel pharmacological treatments and improve clinical outcomes [6–8].

The poliovirus receptor (CD155/PVR), an immunoglobulin superfamily glycoprotein, was initially identified as a mediator of poliovirus entry [9]. Recent studies have expanded its role to include critical functions in cell adhesion, migration, proliferation, contact inhibition, and immune modulation [10, 11]. It is noteworthy that CD155 exhibits relatively low expression levels in normal tissues, yet its expression is significantly upregulated in a range of cancers, including lung, breast, colorectal, and gastric cancers. Moreover, the elevated expression of CD155 is closely associated with poor prognosis [12]. CD155 expression has been shown to positively correlate with tumor size and staging, with significantly higher levels observed in advanced cancers (stages III and IV) compared with early-stage malignancies. In lung cancer, the interaction between CD155 and TIGIT has been demonstrated to directly inhibit the function of natural killer (NK) cells, thereby facilitating tumor immune escape [13]. In breast cancer, CD155 can enhance mitochondrial fatty acid β-oxidation via the CD155-CD96-Src-Stat3-Opa1 signaling axis, thereby promoting the chemotherapy resistance of breast cancer stem cells [14]. In rectal cancer, PITPNC1 can regulate the expression of CD155 via FASN, suppress CD8^+^ T-cell immune function, and enhance radiation resistance in rectal cancer [15]. He et al. discovered that gastric cancer cells can inhibit CD8 T-cell metabolism via CD155/TIGIT signaling, thus suppressing the effector function of CD8 T cells and resulting in hyporesponsive antitumor immunity [16]. In the context of OS, Zhuo et al. demonstrated that overexpression of CD155 significantly enhances the lung metastatic potential of OS cells [17]. Despite emerging insights into CD155’s role in cancer progression, its involvement in OS and the underlying mechanisms remain underexplored.

The Wnt/β-catenin signaling pathway, known for its evolutionary conservation, is implicated in a myriad of cellular processes, including proliferation, differentiation, migration, invasion, and tissue homeostasis, and plays a critical role in tumorigenesis [18, 19]. The interaction between CD155 and the Wnt/β-catenin pathway in the context of malignant progression is not well understood and thus necessitates further investigation.

Troxerutin, a derivative of the naturally occurring flavonoid rutin, is renowned for its extensive pharmacological attributes including antioxidant and anti-inflammatory capabilities. Consequently, it finds widespread clinical application in the management of chronic venous insufficiency and various other vascular ailments [20]. Recent studies have also highlighted its protective effects in Alzheimer’s disease, colorectal cancer, and hepatocellular carcinoma [21]. However, its application and mechanisms in treating OS remain largely unexplored.

In this study, we investigated the role of CD155 in OS and identified potential CD155-targeting drugs through virtual screening. By conducting both in vitro and in vivo experiments, we demonstrated that CD155 significantly contributes to the malignant progression of OS. Through bioinformatics, co-immunoprecipitation (co-IP), and RNA-seq analysis, we established that CD155 enhances SRC activity, activates the PI3K-AKT signaling pathway, and consequently triggers the Wnt/β-catenin pathway. Furthermore, our structure-based virtual screening identified troxerutin as an effective inhibitor of CD155, capable of suppressing OS stemness. These findings suggest that troxerutin holds significant therapeutic promise for targeting CD155 to inhibit OS malignancy, offering a new therapeutic approach for patients with OS.

## Methods

### Human samples

The human OS samples and adjacent normal tissues utilized in this study were approved by the Ethics Committee of The First Affiliated Hospital of Sun Yat-sen University, Guangzhou (approval no. [2021] 755). Informed consent was obtained from all participants. mRNA levels were analyzed in 20 matched tumor and adjacent normal tissues using quantitative real-time polymerase chain reaction (qRT-PCR), while protein expression in 12 paired samples was assessed via Western blot. Additionally, immunohistochemistry was performed on 53 tumor samples to evaluate protein localization and expression levels. This study was conducted in strict adherence to the Declaration of Helsinki and has received approval from the Ethical Review Committee of the First Affiliated Hospital of Sun Yat-sen University.

### Cell culture

OS cell lines SAOS-2, U2OS, MG63, HOS, MNNG/HOS, 143B, SJSA-1, and G292 and the osteoblast cell line hFOB1.19 were acquired from the American Type Culture Collection (ATCC). The methotrexate-resistant U2OS/MTX300 cell line was provided by Dr. M. Serra, Istituti Ortopedici Rizzoli, Bologna, Italy. The primary and metastatic OS cell lines, ZOS and ZOSM, have been previously described [22]. All cell lines were authenticated and cultured following ATCC’s protocols.

### Collection of gene expression datasets

The gene expression data for pan-cancer was sourced from The Cancer Genome Atlas Program (TCGA, https://www.cancer.gov/ccg/research/genome-sequencing/tcga), encompassing 11,123 research specimens. Statistical analysis was performed using the Wilcoxon rank-sum test, and the results are ultimately presented as mean values on the graph.

### The survival curve for pan-cancer

The pan-cancer survival curve was plotted using the Gene Expression Profiling Interactive Analysis 2 (GEPIA2, http://gepia2.cancer-pku.cn/) database. All patients were sorted based on their CD155 expression levels, with the median serving as the cutoff. Subsequently, the patients were divided into two groups (high-CD155 group and low-CD155 group).

### RNA extraction and qRT-PCR

Total RNA was extracted using TRIzol reagent (Invitrogen, USA) and converted to cDNA using the PrimeScript RT reagent kit (Takara, Japan). qRT-PCR was performed using SYBR Green SuperMix (Roche, Switzerland) on an ABI7900HT Fast real-time PCR system (Applied Biosystems, USA), with ACTB serving as the internal control. Primer sequences are detailed in Supplementary Table S1.

### Western blot analysis

Cellular proteins were extracted using radioimmunoprecipitation assay (RIPA) buffer with protease and phosphatase inhibitors (Roche, Switzerland). Protein concentrations were quantified using the Pierce™ BCA protein assay kit (Thermo Fisher Scientific, USA). Equal amounts of proteins were separated by sodium dodecyl sulfate (SDS)-polyacrylamide gel electrophoresis (PAGE), transferred to polyvinylidene fluoride (PVDF) membranes (Millipore), and probed with specific antibodies. Detection was performed using enhanced chemiluminescence (ECL; MeilunBio, China).

The primary antibody used for western blotting and immunohistochemistry assays were as follows: CD155 (CST, 81254), GAPDH (Proteintech, 60004–1-Ig), β-tubulin (Proteintech, 10094–1-AP), β-catenin (Proteintech, 51067–2-AP), histone H3 (CST, 9715), AKT (Proteintech, 60203–2-Ig), p-AKT (Proteintech, 66444–1-Ig), GSK3β (Proteintech, 22104–1-AP), p-GSK3β (Proteintech, 67558–1-Ig), SRC (CST, 2108), p-SRC Tyr 416 (CST, 6943), p-SRC Tyr 527 (CST, 2105), and Ki-67 (CST, 9449).

### Immunohistochemical analysis

Immunostaining was performed on tissue sections, which were scored based on the percentage of positive cells and staining intensity. The overall expression score was calculated by multiplying these two factors. On the basis of the immune response scoring system, the immunohistochemical staining level was quantified. The cutoff for defining high or low expression of CD155 in immunohistochemistry was set at the median immunohistochemical score (*n* = 53), which included 31 patients with high expression of CD155 and 22 patients with low expression of CD155.

### Lentiviral transduction

Lentiviruses packaging CD155 shRNA (targeting sequences: Sh_CD155_a: TTGCAGGTCACATTCTTGCCG, Sh_CD155_c: AATTGTTGTTGGCGTTTCGGG) were obtained from Tsingke Biotech (Beijing, China). OS cells were transduced and selected with puromycin (1 μg/ml) for stable knockdown.

### Cell proliferation and colony formation assays

Cell proliferation was assessed using the CCK-8 assay (Dojindo, Japan), and colony formation capacity was evaluated by plating cells in six-well plates, followed by staining with crystal violet. Both assays were quantified and data are presented as mean ± standard deviation (SD) from three independent experiments.

### Transwell migration and invasion assays

Cell migration and invasion were analyzed using Transwell chambers with or without a Matrigel coating (Corning). Cells were seeded in serum-free medium and allowed to migrate toward medium supplemented with 10% fetal bovine serum (FBS). Migrated cells were stained, imaged, and counted.

### Apoptosis and sphere formation assays

Apoptosis was induced with cisplatin and analyzed by annexin V/propidium iodide (PI) staining followed by flow cytometry. For sphere formation, a total of 1 × 10^3^ cells were cultured in ultralow-adhesion dishes, and following stem-cell-promoting conditions (with SJSA-1 cultured for 14 days and 143B for 7 days), the number of spheres with diameter exceeding 50 μm was enumerated.

### ALDEFLUOR assay

The ALDEFLUOR assay utilizing the ALDEFLUOR kit (Stem Cell Technologies, Vancouver, Canada) was applied to measure the aldehyde dehydrogenase (ALDH) activity of OS cells through fluorescence-activated cell sorting, following the manufacturer’s instructions. The experiment was conducted in triplicate.

### Animal studies

The effect of CD155 knockdown on tumor growth was evaluated in nude mice, following ethical approvals. Mice were injected with OS cells and monitored for tumor development. Tumor volumes were calculated and analyzed statistically. Tumor size was measured in two perpendicular dimensions (D1 and D2). As previously mentioned, tumor volume was calculated using the formula *V* = 4/3*π*[1/4 (*D*_1_ + *D*_2_)]^2^ [23].

### RNA-seq and co-immunoprecipitation

For RNA-seq, libraries were prepared and sequenced, followed by data analysis to identify differentially expressed genes. co-IP was used to study protein interactions, with specific details on antibodies and conditions provided. The primary antibody used for co-IP assays were CD155 (Abcam, ab205304) and SRC (CST, 2108).

### Virtual screening and molecular dynamics

Structure-based virtual screening was conducted using Autodock Vina, targeting CD155 with Food and Drug Administration (FDA)-approved drug libraries to identify potential inhibitors. Pymol/Origin Pro software was used to generate images from molecular dynamics simulations. Subsequently, experiments were conducted to verify the changes in the binding ability between CD155 and SRC after the addition of troxerutin (MedChemExpress, USA; purity ≥ 98.0%).

### Statistical analysis

Data were analyzed using GraphPad Prism 9, with statistical significance set at *p* < 0.05. Results are expressed as mean ± SD from triplicate experiments.

## Results

### CD155 correlates with poor prognosis in OS

**Fig. 1:**
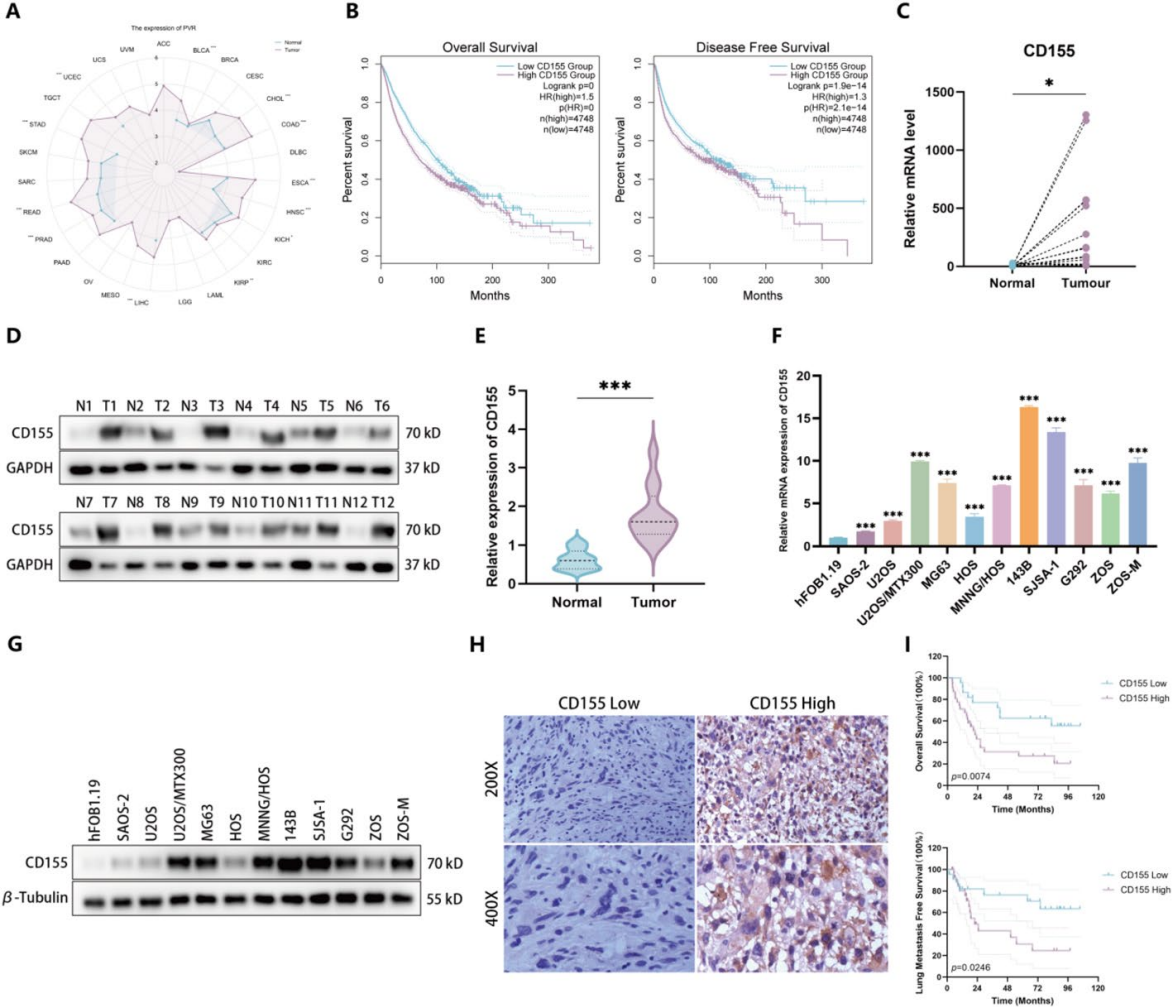
CD155 correlates with poor prognosis in OS. **A** Analysis of the TCGA database reveals that the expression level of CD155 is notably elevated in a range of tumors, in stark contrast to its corresponding normal tissues. **B** Data analysis from the GEPIA2 database indicates that patients with a high level of CD155 expression have a worse prognosis among cancer patients (the dashed line indicates the 95% confidence interval). **C** qRT-PCR was employed to assess the mRNA expression level of CD155 in both human OS tissues and their corresponding normal tissues (*n* = 20). **D** Western blot experiments were performed to quantitatively evaluate the expression levels of CD155 protein in human OS tissues, as well as their corresponding normal tissues, for the purpose of comparative analysis. **E** A grayscale analysis on the outcomes presented in **D**. **F** The expression of CD155 between hFOB1.19 and OS cell lines detected by qRT-PCR. **G** Western blot experiment to measure the CD155 protein levels between hFOB1.19 and OS cell lines. **H** Representative IHC images demonstrating the expression of CD155 in the tumor tissues of OS patients. **I** Kaplan–Meier survival analysis of OS patients stratified by CD155 expression levels (*n* = 53, the dashed line indicates the 95% confidence interval). *p < 0.05; **p < 0.01; ***p < 0.001

Upon analyzing The Cancer Genome Atlas (TCGA) database, we discovered that the expression level of CD155 is notably elevated in the majority of tumors compared with their respective normal tissues (Fig. 1A). An initial analysis using the Gene Expression Profiling Interactive Analysis 2 (GEPIA2) database indicated that high CD155 expression correlates with reduced overall and disease-free survival in patients with cancer (Fig. 1B). Subsequent qRT-PCR analysis of 20 samples from patients with OS revealed significantly higher CD155 mRNA levels in tumor tissues compared with adjacent normal tissues (Fig. 1C). Western blot analyses corroborated these findings, showing increased CD155 protein levels in tumor tissues (Fig. 1D, E). Elevated levels of CD155 mRNA and protein were also observed in various OS cell lines compared with the osteoblast cell line hFOB1.19 (Fig. 1F, G). Immunohistochemical (IHC) analysis of OS clinical specimens further confirmed higher CD155 expression, which was associated with lower overall survival and lung metastasis-free survival rates, as shown by Kaplan–Meier analysis (Fig. 1H, I). Collectively, these data suggest a prognostic role for CD155 in OS, with its overexpression linked to adverse clinical outcomes.

### CD155 enhances malignant phenotypes of OS

**Fig. 2:**
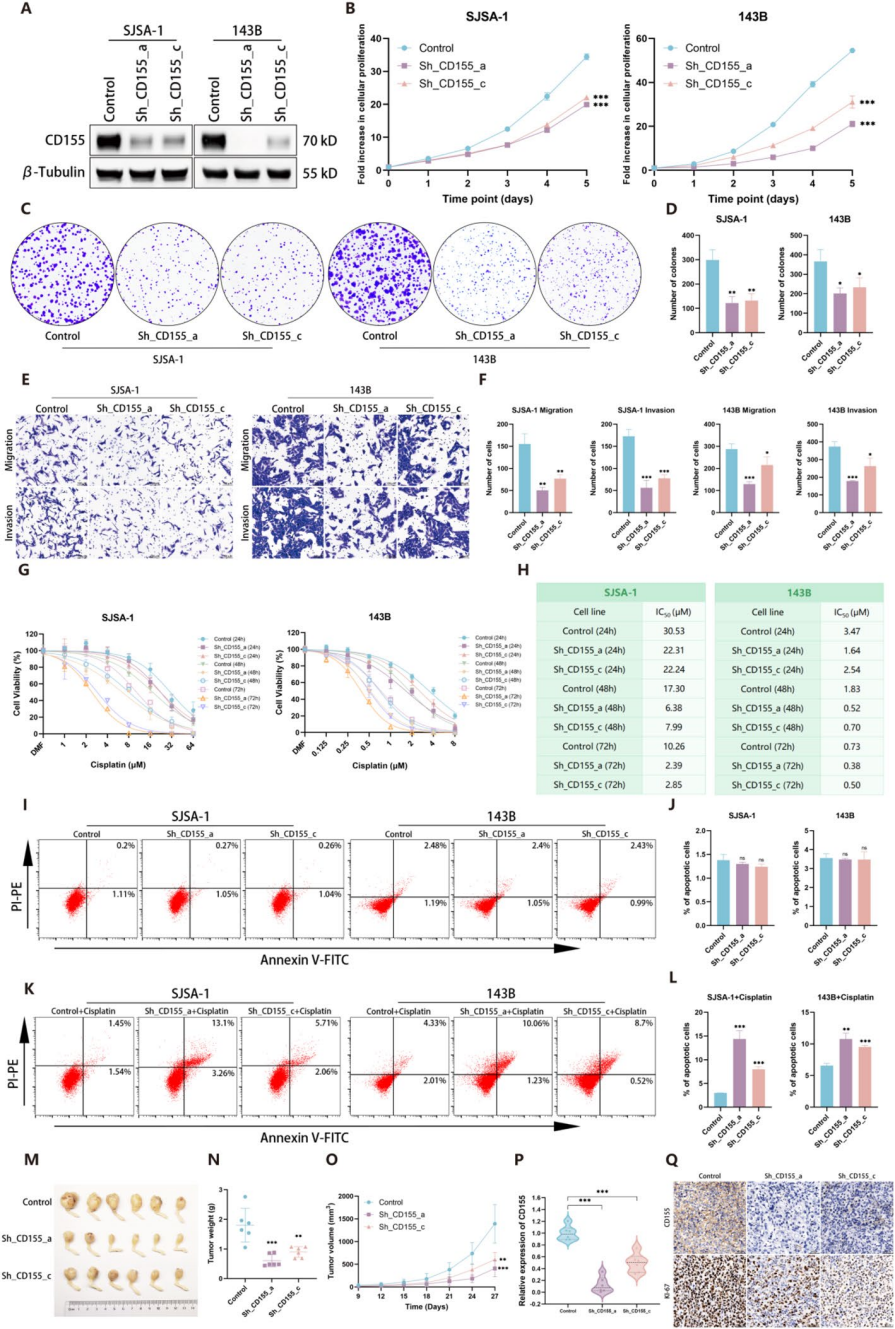
CD155 enhances malignant phenotypes of OS. **A** Western blot is used to assess the levels of CD155 protein in SJSA-1 and 143B cells following the knockdown of CD155. **B**–**D** The CCK-8 assay (**B**) and colony formation assay (**C**, **D**) to assess the proliferative capacity of OS cells following CD155 knockdown. **E**, **F** Utilizing the Transwell assay, the invasion and migration capacities of OS cells were evaluated. **G**, **H** The CCK-8 assay was employed to assess the resistance of OS cells toward cisplatin. **I**, **J** Flow cytometry was employed to assess the apoptotic state of OS cells following knockdown of CD155. **K**, **L** Flow cytometry was utilized to assess the apoptotic status of OS cells following cisplatin treatment (SJSA-1 cells pretreated with 9 μM cisplatin for 48 h and 143B cells pretreated with 1 μM cisplatin for 48 h). **M** Knocking down CD155 can effectively suppress the growth of 143B cells in nude mice. **N** After a period of 27 days, the tumor were extracted and subsequently weighed. **O** The tumor volume was monitored every 3 days to generate a tumor growth curve accordingly. **P** Western blot experiments were performed to assess the expression level of CD155 in 143B cells following tumor formation in nude mice, followed by grayscale analysis. **Q** IHC staining of tumor sections was performed using CD155 and KI-67 antibodies. *p < 0.05; **p < 0.01; ***p < 0.001

To explore CD155’s functional role in OS, we generated stable CD155 knockdown in SJSA-1 and 143B cells using two shRNA sequences (Sh_CD155_a and Sh_CD155_c) (Fig. 2A). Knockdown of CD155 significantly reduced proliferation (Fig. 2B) and colony formation (Fig. 2C, D) in these cell lines. Transwell assays also showed reduced migration and invasion capabilities following CD155 suppression (Fig. 2E, F). Additionally, reduced CD155 expression decreased resistance to cisplatin, a primary chemotherapeutic agent for OS (Fig. 2G–L) [24]. Building on in vitro results, 143B cells with CD155 knockdown were implanted into the tibias of nude mice. This led to significantly suppressed OS cell growth in vivo (Fig. 2M–O). Subsequently, Western blot experiments were performed to assess the alterations in the expression level of CD155 following tumor nude mice (Fig. 2P). IHC analysis showed a positive correlation between CD155 and Ki-67, a proliferation marker, in OS (Fig. 2Q). These findings indicate that CD155 downregulation hampers OS cell proliferation, metastasis, and chemoresistance.

### CD155 facilitates OS cell stemness

**Fig. 3:**
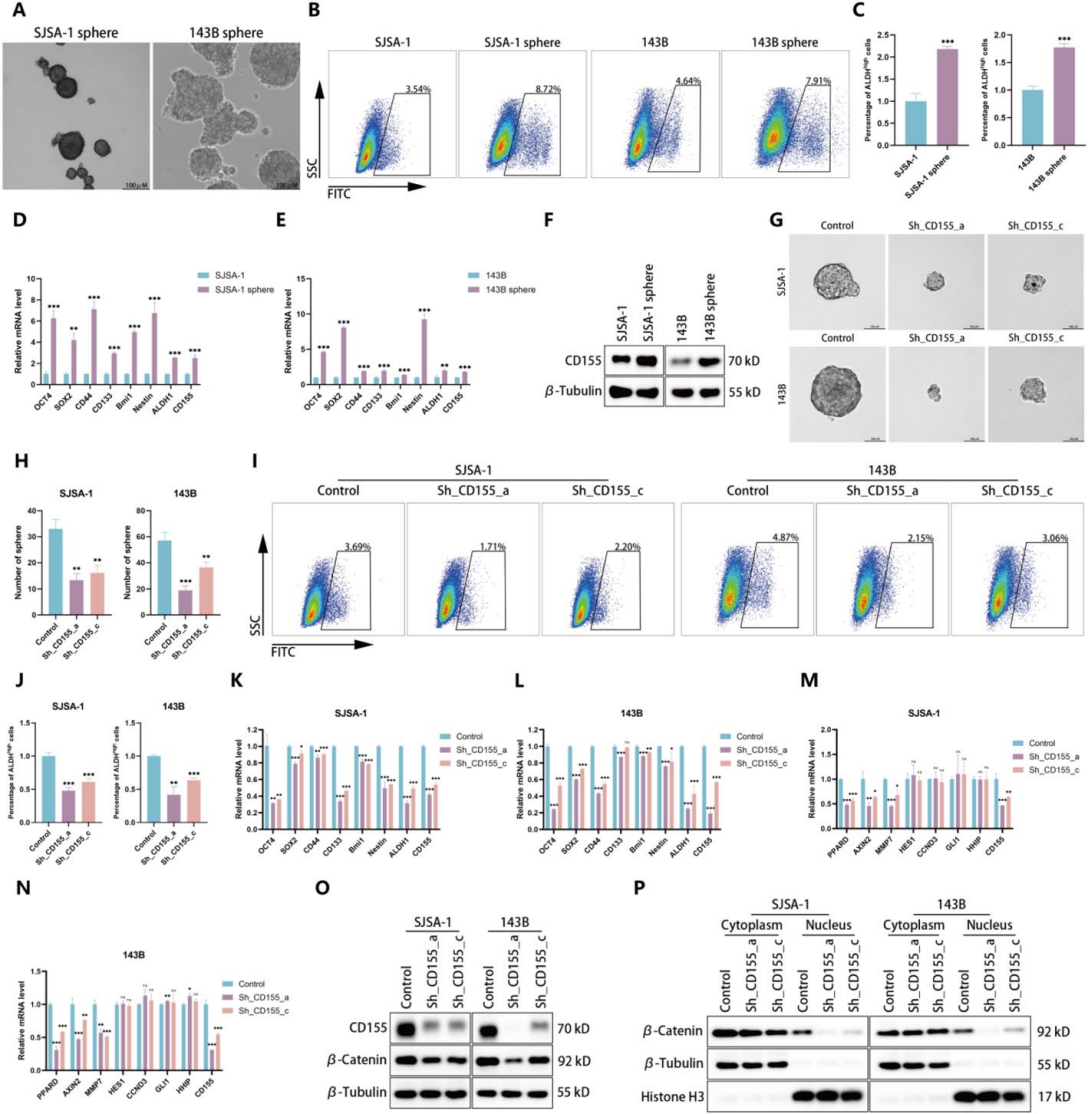
CD155 facilitates OS cell stemness. **A** Representative images depicting CSCs isolated from SJSA-1 and 143B cells. **B**, **C** The activity of ALDH in CSCs was measured using fluorescence-activated cell sorting analysis. Representative images and quantitative data of ALDH activity results are presented. **D**, **E** Utilize the qRT-PCR experiment to assess the expression of stemness markers and CD155 in CSCs cells. **F** Determine the protein expression level of CD155 in CSCs by western blotting. **G** Representative images of in vitro sphere formation assay conducted on control cells and CD155-knockdown OS cells. **H** The quantity of sphere in the in vitro sphere formation assay conducted on CD155-knockdown OS cells and their respective control cells. **I**, **J** The activity of ALDH in CD155-knockdown SJSA-1 and 143B cells was measured using fluorescence-activated cell sorting analysis. Representative images and quantitative data of ALDH activity results are presented. **K**, **L** qRT-PCR was utilized to assess the expression levels of stemness markers in CD155-knockdown SJSA-1 and 143B cells. **M**, **N** Utilize qRT-PCR to assess the mRNA expression levels of target genes involved in the Wnt pathway (PPARD, AXIN2, and MMP7), Notch pathway (HES1 and CCND3) and Hedgehog pathway (GLI1 and HHIP) in both control cells and CD155 knockdown OS cells. **O** Detection of alterations in β-catenin protein levels in OS cells following CD155 knockdown by western blotting. **P** Detection of alterations in β-catenin nuclear protein levels in OS cells following CD155 knockdown via western blotting. *p < 0.05; **p < 0.01; ***p < 0.001

Investigations into CD155’s role in cancer stemness showed that CD155 expression and ALDH activity were elevated in sarcospheres derived from SJSA-1 and 143B cells, which exhibit cancer stem cell (CSC) traits, compared with parental cells (Fig. 3A–F). Knockdown of CD155 results in a reduction in the size and number of spheroids (Fig. 3G, H), decreased ALDH activity (Fig. 3I, J), and reduced expression of cancer stemness markers (Fig. 3K, L). Examination of CSC pathways revealed significant downregulation of Wnt pathway target genes following CD155 knockdown (Fig. 3M, N) [25–28]. Consistent with this finding, β-catenin expression and nuclear localization were reduced post-CD155 knockdown (Fig. 3O, P), suggesting that CD155 supports OS stemness primarily through the Wnt/β-catenin pathway.

### Overexpression of CD155 augments malignant OS phenotypes

**Fig. 4:**
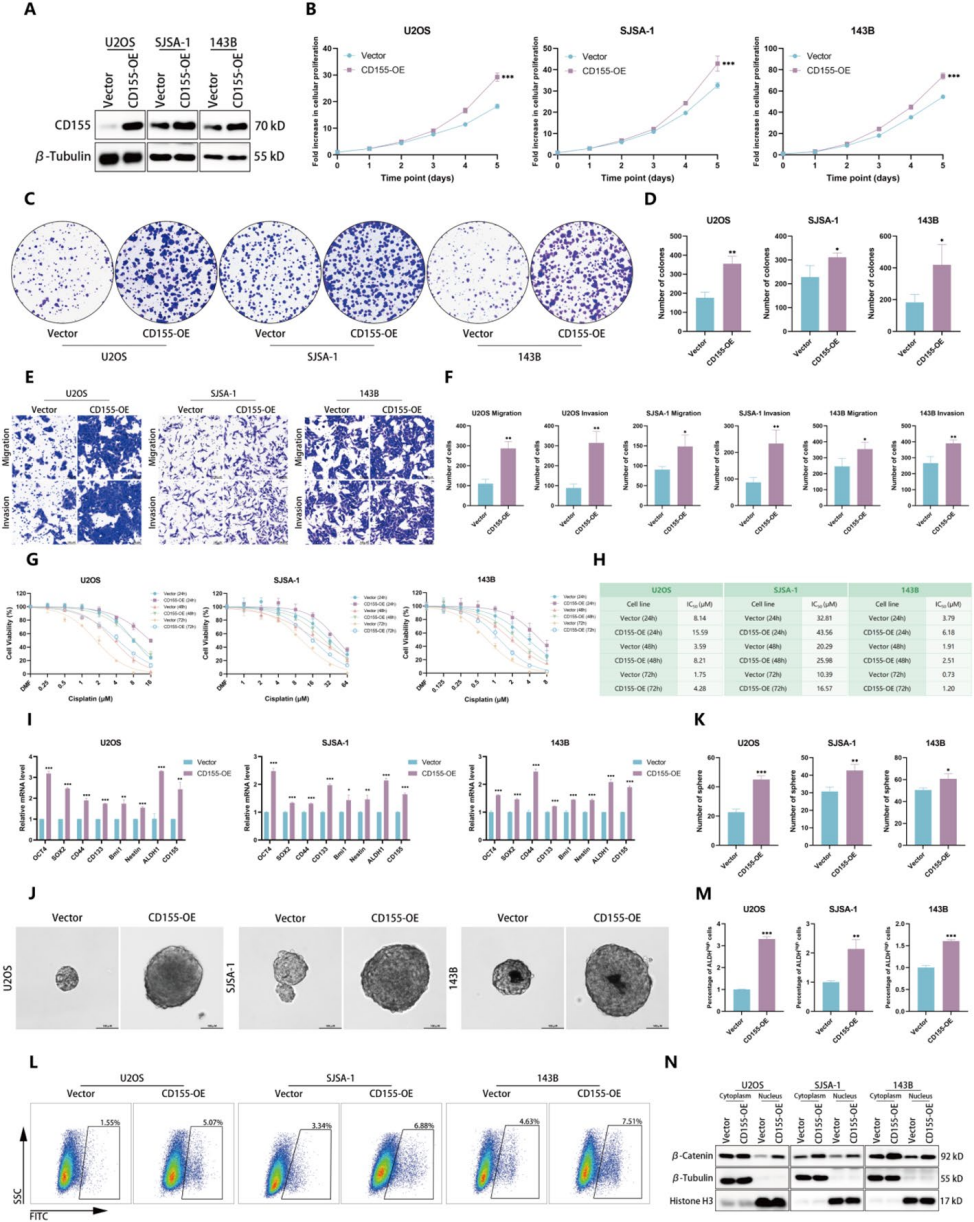
Overexpression of CD155 augments malignant OS phenotypes. **A** Western blot analysis was employed to assess the levels of CD155 protein in U2OS, SJSA-1, and 143B cells following overexpression of CD155. **B**–**D** CCK-8 assay (**B**) and colony formation assay (**C**, **D**) were employed to assess the proliferative capacity of OS cells following the overexpression of CD155. **E**, **F** The Transwell assay was used to assess the invasive and migratory capabilities of OS cells. **G**, **H** The CCK-8 assay was employed to assess the resistance of OS cells toward cisplatin. **I** qRT-PCR was utilized to assess the expression levels of stemness markers in U2OS, SJSA-1, and 143B cells following the overexpression of CD155. **J** Representative images of in vitro sphere formation assays conducted on control cells and CD155-overexpressing OS cells. **K** The quantity of sphere in the in vitro sphere formation assay conducted on CD155-overexpressing OS cells and their respective control cells. **L**, **M** The activity of ALDH in CD155-overexpressing OS cells was measured using fluorescence-activated cell sorting analysis. Representative images and quantitative data of ALDH activity results are presented. **N** Detection of alterations in β-catenin nuclear protein levels in OS cells following the overexpression of CD155 via western blotting. *p < 0.05; **p < 0.01; ***p < 0.001

To further validate CD155’s role, we overexpressed CD155 in U2OS, SJSA-1, and 143B cells, which enhanced proliferation, metastatic potential, and drug resistance (Fig. 4A–H). In addition, overexpression of CD155 results in an elevation of cancer stemness markers, an increase in the number and size of spheroids, as well as an elevation in ALDH activity (Fig. 4I–M). Increased β-catenin nuclear localization upon CD155 overexpression (Fig. 4N) corroborated its role in activating the Wnt/β-catenin pathway, reinforcing CD155’s potential to amplify OS malignancy.

### β-Catenin promotes malignant phenotypes in OS in vitro

**Fig. 5:**
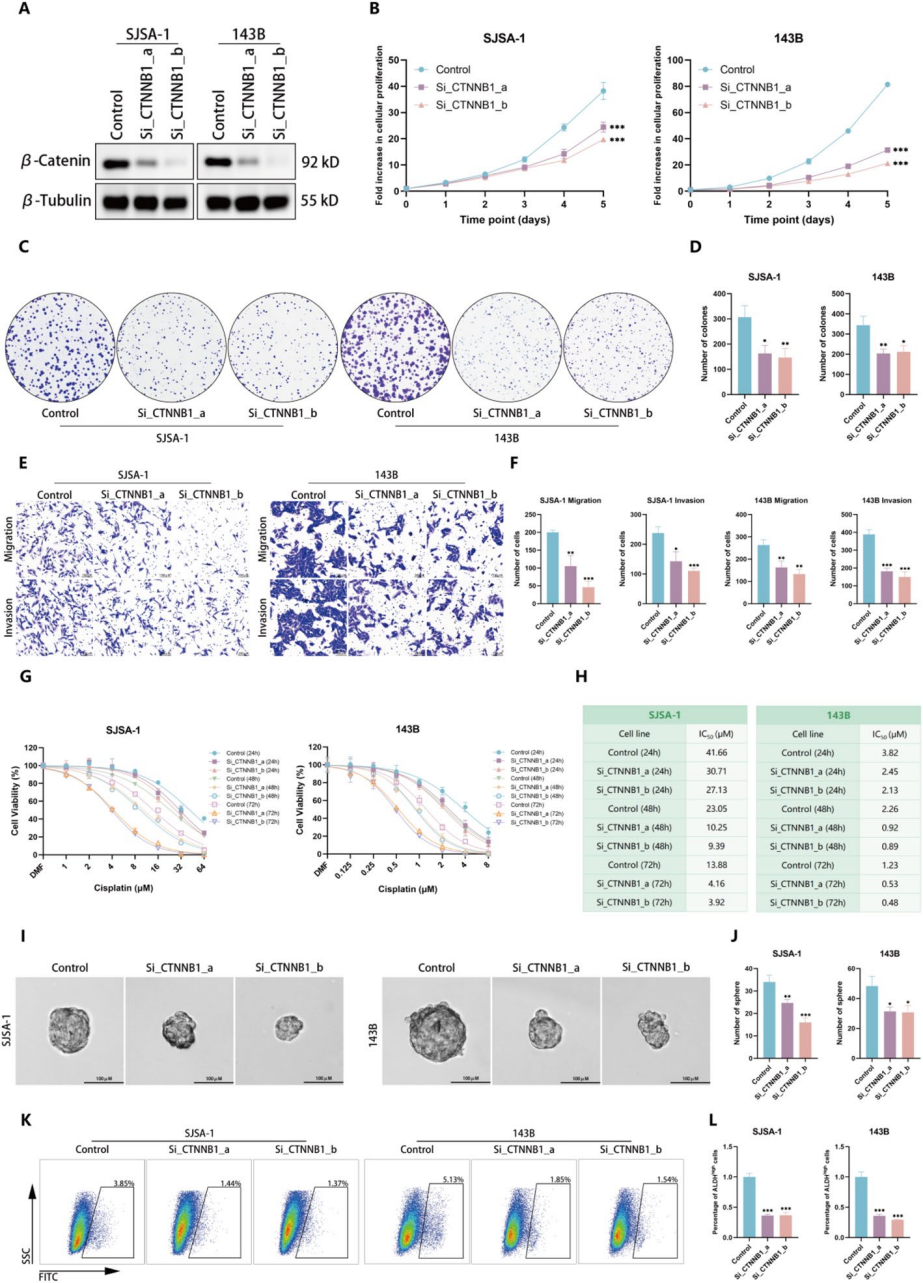
β-Catenin promotes malignant phenotypes in OS in vitro. **A** Western blot is used to assess the levels of β-catenin protein in SJSA-1 and 143B cells following the knock-down of CTNNB1. **B**–**D** The CCK-8 assay (**B**) and colony formation assay (**C**, **D**) were employed to assess the proliferative capacity of OS cells following CTNNB1 knockdown. **E**, **F** Utilizing the Transwell assay, the invasion and migration capacities of OS cells were evaluated. **G**, **H** The CCK-8 assay was employed to assess the resistance of OS cells towards cisplatin. **I** Representative images of in vitro sphere formation assay conducted on control cells and CTNNB1-knockdown OS cells. **J** The quantity of spheres in the in vitro sphere formation assay conducted on CTNNB1-knockdown OS cells and their respective control cells. **K**, **L** The activity of ALDH in CTNNB1-knockdown OS cells was measured using fluorescence-activated cell sorting analysis. Representative images and quantitative data of ALDH activity results are presented. *p < 0.05; **p < 0.01; ***p < 0.001

Building on our understanding that CD155 influences OS progression through the Wnt/β-catenin pathway, we investigated the pathway’s role by silencing β-catenin (CTNNB1) in SJSA-1 and 143B cell lines (Fig. 5A). Post-knockdown, a notable reduction in proliferation, metastasis potential, and chemoresistance was observed (Fig. 5B–H). Furthermore, knockdown of CTNNB1 results in a reduction in the size and number of spheroids and decreased ALDH activity (Fig. 5I–L). To gain a deeper understanding of the Wnt/β-catenin pathway’s role in OS, we treated OS cells with HLY78, a recognized activator of the Wnt/β-catenin pathway. HLY78 specifically targets the DIX domain of Axin, strengthening its interaction with LRP6, thereby facilitating LRP6 phosphorylation and subsequent Wnt signaling transduction [29–31]. Notably, HLY78 treatment resulted in a significant increase in the nuclear localization of β-catenin, effectively enhancing cellular proliferation, metastasis, and drug resistance capabilities (Supplementary Fig. S1A-H). Furthermore, upon the introduction of HLY78, we observed a remarkable surge in both the number and size of spheroids (Supplementary Fig. S1I, J). Finally, we introduced HLY78 into cells with downregulated β-catenin to observe cellular changes, yet these alterations appeared to be rather subtle (Supplementary Fig. S2A-F). We hypothesize that HLY78 inhibits the degradation of β-catenin by specifically targeting the DIX domain of Axin and enhancing the interaction between Axin and LRP6. Nevertheless, in β-catenin knockdown cells, the protein translation of β-catenin is significantly suppressed, thereby limiting observable recovery effects. In conclusion, the Wnt pathway plays a pivotal role in the malignancy of OS.

### CD155 facilitates OS malignancy via Wnt/β-catenin activation

**Fig. 6:**
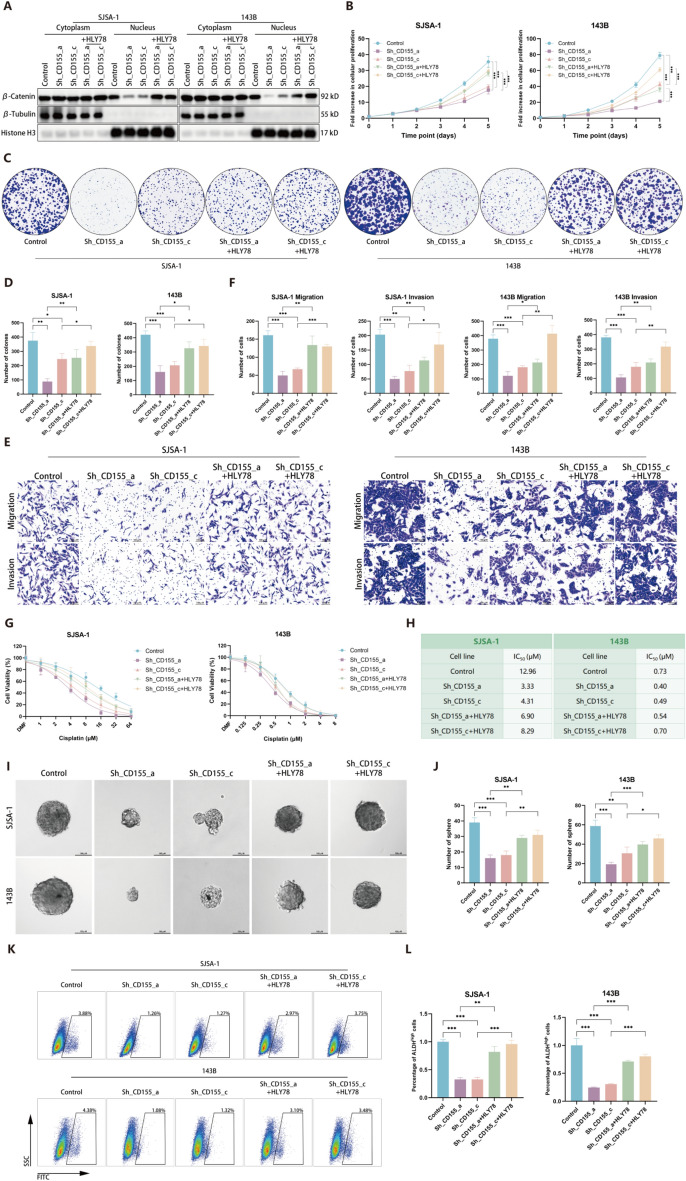
CD155 facilitates OS malignancy via Wnt/β-catenin activation. **A** The nuclear translocation of β-Ccatenin in OS cells with knocked-down CD155 was evaluated using the western blot following the addition of HLY78. (**B**–**D**) The CCK-8 (**B**) assay and colony formation assay (**C**, **D**) were employed to assess the proliferative capacity of OS cells upon the addition of HLY78 to cells with downregulated CD155 expression. **E**, **F** Utilizing the Transwell assay, the invasion and migration capacities of OS cells were evaluated. **G**, **H** The CCK-8 assay was employed to assess the resistance of OS cells toward cisplatin. **I** Representative images of the in vitro sphere formation assay. **J** The quantity of spheres in the in vitro sphere formation assay. **K**, **L** The activity of ALDH in OS cells was measured using fluorescence-activated cell sorting analysis. Representative images and quantitative data of ALDH activity results are presented. *p < 0.05; **p < 0.01; ***p < 0.001

To ascertain whether the augmentation of malignancy by CD155 is facilitated through the Wnt/β-catenin pathway, we administered HLY78 to OS cells with downregulated CD155. The treatment with HLY78 significantly reinstated the nuclear localization of β-catenin in CD155-knockdown cells, effectively restoring their proliferative, metastatic, and drug-resistant capabilities (Fig. 6A–H). Moreover, upon the addition of HLY78, both the number and size of the observed spheres, as well as ALDH activity, were significantly restored (Fig. 6I–L). These findings underscore the pivotal role of CD155 in augmenting OS malignancy by potentiation of the Wnt/β-catenin pathway.

### CD155 activates Wnt/β-catenin signaling via the PI3K-AKT pathway

**Fig. 7:**
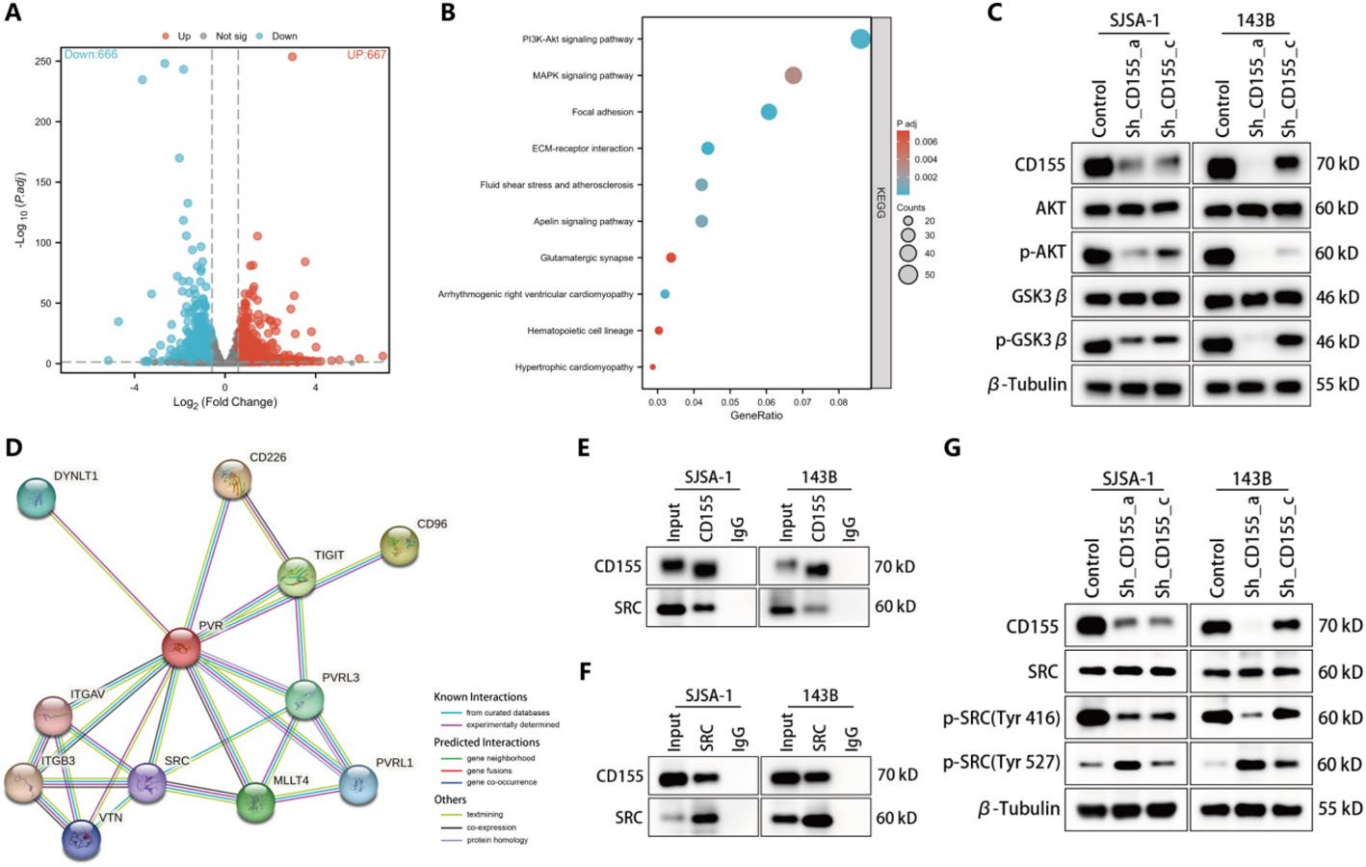
CD155 activates Wnt/β-catenin signaling via the PI3K-AKT pathway. **A** The volcano plot illustrates the increase and decrease in the number of genes in 143B cells following CD155 knockdown, in comparison with control cells. **B** KEGG enrichment analysis revealed that the PI3K-AKT signaling pathway exhibited the most significant alteration. **C** Western blot was utilized to assess alterations in the protein levels of phosphorylated AKT and phosphorylated GSK3β in OS cells with CD155 knockdown. **D** Proteins potentially interacting with CD155 (PVR) predicted by the STRING database. **E**, **F** The interaction between CD155 and SRC in SJSA-1 and 143B cells was examined through a co-IP experiment. **G** Western blot was utilized to assess alterations in the protein levels of phosphorylated SRC in OS cells following the knockdown of CD155

Further exploration using RNA-seq identified significant gene expression changes post-CD155 knockdown in 143B cells. Kyoto Encyclopedia of Genes and Genomes (KEGG) pathway analysis of differentially expressed genes highlighted the PI3K-AKT pathway as significantly enriched (Fig. 7A, B). Concordant with previous studies, we observed that activation of this pathway promotes β-catenin’s nuclear translocation by phosphorylating GSK3β [32]. Consequently, reduced phosphorylation levels of AKT and GSK3β were confirmed post-CD155 knockdown, supporting the involvement of this pathway in CD155-mediated Wnt/β-catenin activation (Fig. 7C). Additionally, using the Search Tool for the Retrieval of Interacting Genes/Proteins (STRING) database, we identified potential CD155 (PVR) interacting proteins, emphasizing SRC owing to its known role in activating the PI3K-AKT pathway (Fig. 7D) [33, 34]. Co-IP assays confirmed the interaction between CD155 and SRC, and specific alteration in phosphorylation states of SRC at Tyr 416 further delineated this mechanism (Fig. 7E–G).

### Troxerutin targets CD155 to mitigate OS stemness

**Fig. 8:**
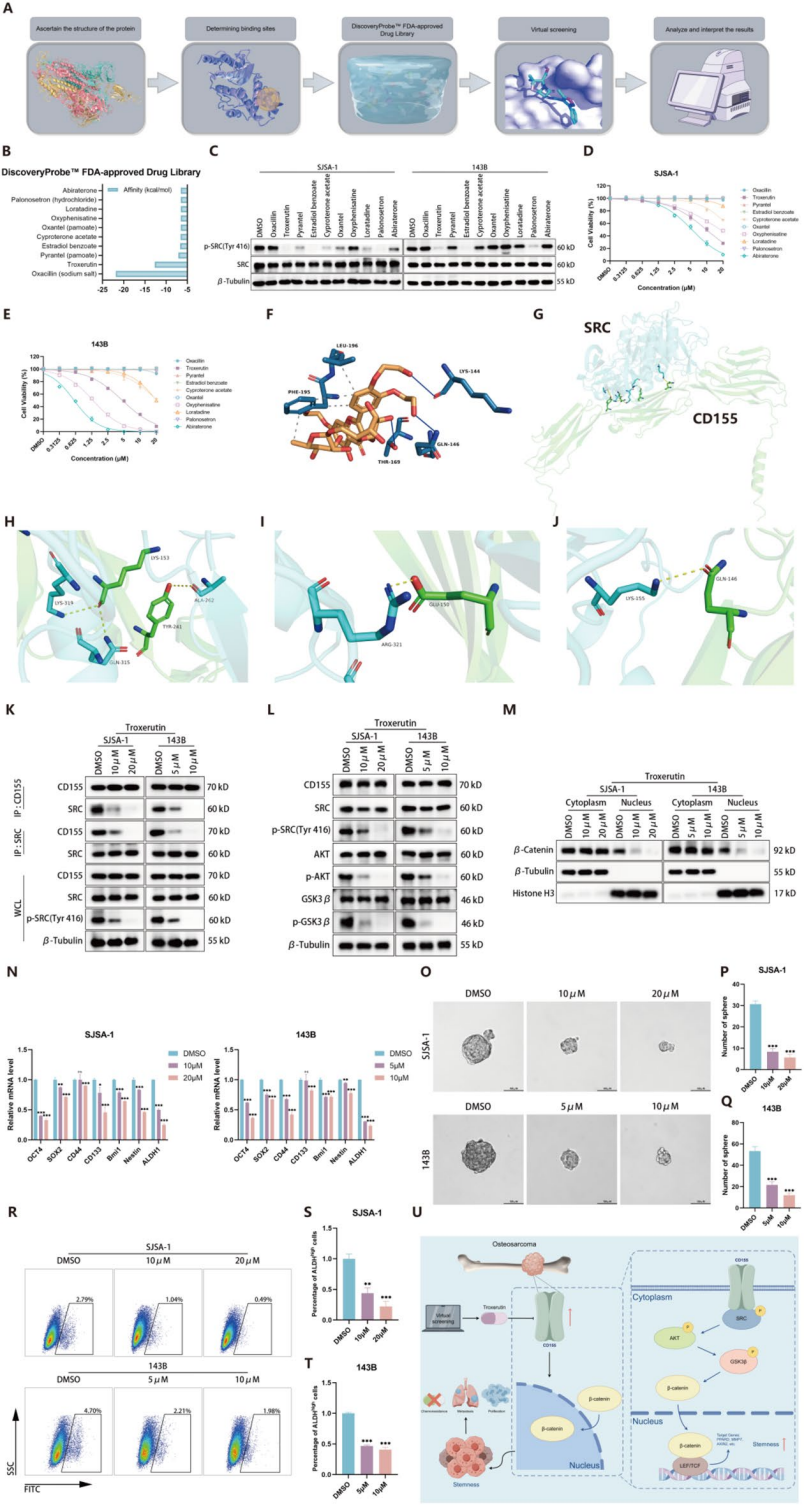
Troxerutin targets CD155 to mitigate OS stemness. **A** Illustrative workflow diagram for the screening of potential therapeutics directed against CD155. **B** The top ten drugs, ranked based on affinity, with lower values indicating stronger affinity. **C** Western blot was utilized to assess the alterations in the phosphorylation levels of SRC following the introduction of the top ten drugs, ranked by affinity, into SJSA-1 and 143B cells. **D**, **E** The CCK-8 assay is utilized to assess the resistance of OS cells to the top ten drugs ranked by affinity. **F** The interaction diagram between CD155 and troxerutin, depicting hydrogen bonding with solid blue lines and hydrophobic interaction with dashed gray lines. **G** Hydrogen bonding diagram of CD155 and SRC (overall). **H**–**J** Hydrogen bonding diagram of CD155 and SRC (details). **K** The co-IP experiment was conducted to investigate the interaction between CD155 and SRC in SJSA-1 and 143B cells following troxerutin treatment. **L** Western blot analysis was employed to assess alterations in the phosphorylated SRC, AKT, and GSK3β protein levels within OS cells following troxerutin treatment. **M** Western blot is utilized to assess the nuclear translocation of β-catenin in OS cells following troxerutin treatment. **N** qRT-PCR was utilized to assess the expression levels of stemness markers in SJSA-1 and 143B cells following troxerutin treatment. **O** Representative images of the in vitro sphere formation assay. **P**, **Q** The quantity of spheres in the in vitro sphere formation assay. **R**–**T** The activity of ALDH in OS cells was measured using fluorescence-activated cell sorting analysis. Representative images and quantitative data of ALDH activity results are presented. **U** A refined model demonstrating troxerutin’s suppressive effect on OS stemness via the CD155/SRC/β-catenin signaling axis. *p < 0.05; **p < 0.01; ***p < 0.001

To identify potential CD155-targeting drugs, a structure-based virtual screening was performed, ranking drugs on the basis of affinity to CD155 (Fig. 8A, B). Introduction of the top candidates into OS cell lines revealed that troxerutin, estradiol benzoate, and palonosetron decreased phosphorylated SRC levels, with troxerutin showing distinct OS-suppressive properties (Fig. 8C–E). Furthermore, we conducted molecular docking. Initially, we ascertained the interaction between CD155 and troxerutin (Fig. 8F), followed by determining the interaction between CD155 and SRC (Fig. 8G–J; Supplementary Tables S4, 5). Subsequently, we employed molecular dynamics simulation technology to examine the conformational changes, binding free energy variations, and the altered contributions of each residue to the binding free energy between CD155 and SRC upon the addition of troxerutin (Supplementary Fig. S3A–M; Supplementary Figs. S6–8). We conducted experiments to determine whether troxerutin specifically suppressed the interaction between CD155 and SRC. The experimental findings revealed a marked reduction in the binding capacity of CD155 and SRC upon the introduction of troxerutin into OS cells (Fig. 8K). Detailed analysis confirmed troxerutin’s efficacy in reducing downstream targets of CD155, including phosphorylated SRC, AKT, and GSK3β, which correlated with diminished β-catenin nuclear translocation (Fig. 8L, M). The sphere formation assay and ALDEFLUOR assay further confirmed that the cancer stemness markers, number and size of spheres, and ALDH activity were significantly reduced in troxerutin-treated cells, consolidating their targeting of the CD155/SRC/β-catenin axis to inhibit OS stemness (Fig. 8N–U).

## Discussion

OS remains the most common primary bone tumor among children and adolescents [35]. Despite advancements in multidrug chemotherapy and surgical interventions over the past three decades, which have notably improved the 5-year survival rate for OS patients [1], the development of new treatment options has stagnated in recent years [36, 37]. This underscores the critical need for more effective therapeutic interventions. Our study highlights a significant association between elevated CD155 expression and the malignant progression of OS, suggesting that targeting CD155 could be a promising therapeutic strategy, given its correlation with poor prognosis in patients with OS.

Traditionally, CD155 is minimally expressed in endothelial and immune cells [38, 39] but is abnormally overexpressed in various tumor types [40–42]. Although recognized as an immune regulatory molecule in diverse cancers [43, 44], the specific role of CD155 in OS has been less clear. We discovered that cells originating from the same source and expressing elevated levels of CD155 tend to possess greater malignant potential. For instance, when comparing U2OS with its methotrexate-resistant variant, U2OS/MTX300, the latter exhibited a stronger malignant potential. Similarly, among HOS, MNNG/HOS, and 143B cells, MNNG/HOS and 143B demonstrated superior proliferative and metastatic capabilities [45]. Furthermore, in comparing ZOS and ZOS-M cells, ZOS-M, an OS cell isolated from a metastatic site, exhibited greater malignant potential [22]. Moreover, our research findings have clarified the significant role of CD155 in OS malignancy, revealing the underlying mechanism by which CD155 enhances SRC phosphorylation, subsequently facilitating the phosphorylation of AKT and GSK3β. Prior literature has documented that GSK3β functions as a negative regulator of β-catenin, and its phosphorylation leads to inactivation [46]. Consequently, in cells with reduced CD155 levels, the phosphorylation of GSK3β diminishes, resulting in enhanced degradation of β-catenin. This, in turn, reduces the nuclear translocation of β-catenin, thereby suppressing the stemness and malignant progression of OS cells.

The concept of CSCs—cells within a tumor possessing the capabilities of self-renewal and driving malignancy—is increasingly recognized as a fundamental mechanism underlying therapeutic resistance and recurrence [47–49]. The presence of such CSCs in OS poses significant challenges for effective treatment [50]. Directly targeting CSCs or inhibiting their stemness features could thus represent a viable approach to improving outcomes for patients with OS. In our study, suppression of CD155 effectively reduced the stemness attributes of OS cells, supporting the therapeutic potential of CD155 as a target.

Interest has been growing in pathways that maintain tumor stemness, with significant focus on the Wnt, Notch, and Hedgehog pathways [26, 27]. Our research unequivocally demonstrates that suppressing CD155 can effectively attenuate the Wnt/β-catenin signaling pathway, subsequently mitigating the stem cell properties of OS. This discovery concurs with prior research findings, suggesting that inhibiting this pathway can diminish the malignancy of OS [51]. Furthermore, our experimental validation reveals that, upon overexpression of CD155, there is a marked increase in the nuclear translocation of β-catenin, which subsequently potentiates the stem cell characteristics in OS. Notably, this phenomenon appears more pronounced in OS cells (U2OS) with low CD155 expression.

Further, we demonstrated that β-catenin knockdown reduces OS malignancy, and the addition of HLY78, a β-catenin pathway activator, can partially reverse the reduction in malignancy in OS cells with suppressed CD155 expression. These results suggest that targeting the β-catenin pathway via CD155 manipulation could be an effective strategy.

Our structure-based virtual screening identified several compounds potentially targeting CD155, with troxerutin standing out through experimental validation. Known for its antioxidant, anti-inflammatory, and other protective properties [52], the role of troxerutin in tumor treatment had been less explored. Our research has confirmed that troxerutin effectively targets the CD155/SRC/β-catenin axis, thereby inhibiting the stemness of OS cells. We hope that this finding will pave a novel path for the treatment of OS.

## Conclusions

Our research demonstrates that CD155 interacts with and activates SRC, leading to downstream activation of AKT and GSK3β, and triggering the Wnt/β-catenin signaling pathway. This cascade enhances the stemness of OS cells, contributing to their malignancy and resilience to treatments. Troxerutin emerges as a promising therapeutic candidate, offering a new potential avenue for the treatment of OS.

## Supplementary Information

Additional file 1.

Additional file 2.

Additional file 3.

Additional file 4.

Additional file 5.

Additional file 6.

Additional file 7.

Additional file 8.

Additional file 9.

## Data Availability

The TCGA database (https://www.cancer.gov/ccg/research/genome-sequencing/tcga) was utilized to analyze the expression levels of CD155 in tumors and their corresponding normal tissues. The GEPIA2 database (http://gepia2.cancer-pku.cn/) was utilized to conduct an analysis of patients exhibiting high and low expression levels of CD155. The STRING database (https://version-11-5.string-db.org/) was utilized to conduct an analysis of the potential interacting proteins associated with CD155.

## Abbreviations

OS: Osteosarcoma
ATCC: American type culture collection
co-IP: Co-immunoprecipitation
TCGA: The Cancer Genome Atlas
GEPIA2: Gene expression profiling interactive analysis 2
IHC: Immunohistochemical
CSC: Cancer stem cell
KEGG: Kyoto Encyclopedia of Genes and Genomes
STRING: Search tool for the retrieval of interacting genes/proteins

## References

[1] Biermann JS, Adkins D, Benjamin R, Brigman B, Chow W, Conrad EU 3rd, et al. Bone cancer. J Natl Compr Canc Netw. 2007;5(4):420–37. 17442233 10.6004/jnccn.2007.0037

[2] Valery PC, Laversanne M, Bray F. Bone cancer incidence by morphological subtype: a global assessment. Cancer Causes Control. 2015;26(8):1127–39. 26054913 10.1007/s10552-015-0607-3

[3] Mirabello L, Troisi RJ, Savage SA. International osteosarcoma incidence patterns in children and adolescents, middle ages and elderly persons. Int J Cancer. 2009;125(1):229–34. 19330840 10.1002/ijc.24320 PMC3048853

[4] Kansara M, Teng MW, Smyth MJ, Thomas DM. Translational biology of osteosarcoma. Nat Rev Cancer. 2014;14(11):722–35. 25319867 10.1038/nrc3838

[5] Kim HJ, Lee SG, Kim YJ, Park JE, Lee KY, Yoo YH, et al. Cytoprotective role of autophagy during paclitaxel-induced apoptosis in Saos-2 osteosarcoma cells. Int J Oncol. 2013;42(6):1985–92. 23563171 10.3892/ijo.2013.1884

[6] Ottaviani G, Jaffe N. The epidemiology of osteosarcoma. Cancer Treat Res. 2009;152:3–13. 20213383 10.1007/978-1-4419-0284-9_1

[7] Smrke A, Anderson PM, Gulia A, Gennatas S, Huang PH, Jones RL. Future directions in the treatment of osteosarcoma. Cells. 2021. 10.3390/cells10010172. 33467756 10.3390/cells10010172 PMC7829872

[8] Daw NC, Chou AJ, Jaffe N, Rao BN, Billups CA, Rodriguez-Galindo C, et al. Recurrent osteosarcoma with a single pulmonary metastasis: a multi-institutional review. Br J Cancer. 2015;112(2):278–82. 25422914 10.1038/bjc.2014.585 PMC4453448

[9] Yoshikawa K, Ishida M, Yanai H, Tsuta K, Sekimoto M, Sugie T. Immunohistochemical analysis of CD155 expression in triple-negative breast cancer patients. PLoS ONE. 2021;16(6):e0253176. 34115802 10.1371/journal.pone.0253176 PMC8195407

[10] Li YC, Zhou Q, Song QK, Wang RB, Lyu S, Guan X, et al. Overexpression of an immune checkpoint (CD155) in breast cancer associated with prognostic significance and exhausted tumor-infiltrating lymphocytes: a cohort study. J Immunol Res. 2020;2020:3948928. 32411795 10.1155/2020/3948928 PMC7201814

[11] Bowers JR, Readler JM, Sharma P, Excoffon K. Poliovirus receptor: more than a simple viral receptor. Virus Res. 2017;242:1–6. 28870470 10.1016/j.virusres.2017.09.001 PMC5650920

[12] Sun Y, Luo J, Chen Y, Cui J, Lei Y, Cui Y, et al. Combined evaluation of the expression status of CD155 and TIGIT plays an important role in the prognosis of LUAD (lung adenocarcinoma). Int Immunopharmacol. 2020;80:106198. 31954274 10.1016/j.intimp.2020.106198

[13] Wu JW, Liu Y, Dai XJ, Liu HM, Zheng YC, Liu HM. CD155 as an emerging target in tumor immunotherapy. Int Immunopharmacol. 2024;131:111896. 38518596 10.1016/j.intimp.2024.111896

[14] Li J, Xia Q, Di C, Li C, Si H, Zhou B, et al. Tumor cell-intrinsic CD96 mediates chemoresistance and cancer stemness by regulating mitochondrial fatty acid beta-oxidation. Adv Sci. 2023;10(7):e2202956. 10.1002/advs.202202956 PMC9982582 36581470

[15] Liang J, Liao L, Xie L, Tang W, Yu X, Lu Y, et al. PITPNC1 suppress CD8(+) T cell immune function and promote radioresistance in rectal cancer by modulating FASN/CD155. J Transl Med. 2024;22(1):117. 38291470 10.1186/s12967-024-04931-3 PMC10826121

[16] He W, Zhang H, Han F, Chen X, Lin R, Wang W, et al. CD155T/TIGIT signaling regulates CD8(+) T-cell metabolism and promotes tumor progression in human gastric cancer. Cancer Res. 2017;77(22):6375–88. 28883004 10.1158/0008-5472.CAN-17-0381

[17] Zhuo B, Li Y, Gu F, Li Z, Sun Q, Shi Y, et al. Overexpression of CD155 relates to metastasis and invasion in osteosarcoma. Oncol Lett. 2018;15(5):7312–8. 29725446 10.3892/ol.2018.8228 PMC5920504

[18] Perugorria MJ, Olaizola P, Labiano I, Esparza-Baquer A, Marzioni M, Marin JJG, et al. Wnt-beta-catenin signalling in liver development, health and disease. Nat Rev Gastroenterol Hepatol. 2019;16(2):121–36. 30451972 10.1038/s41575-018-0075-9

[19] Zhao H, Ming T, Tang S, Ren S, Yang H, Liu M, et al. Wnt signaling in colorectal cancer: pathogenic role and therapeutic target. Mol Cancer. 2022;21(1):144. 35836256 10.1186/s12943-022-01616-7 PMC9281132

[20] Ahmadi Z, Mohammadinejad R, Roomiani S, Afshar EG, Ashrafizadeh M. Biological and therapeutic effects of troxerutin: molecular signaling pathways come into view. J Pharmacopuncture. 2021;24(1):1–13. 33833895 10.3831/KPI.2021.24.1.1 PMC8010425

[21] Bianchi M, Canavesi R, Aprile S, Grosa G, Del Grosso E. Troxerutin, a mixture of O-hydroxyethyl derivatives of the natural flavonoid rutin: chemical stability and analytical aspects. J Pharm Biomed Anal. 2018;150:248–57. 29258044 10.1016/j.jpba.2017.12.018

[22] Zou CY, Wang J, Shen JN, Huang G, Jin S, Yin JQ, et al. Establishment and characteristics of two syngeneic human osteosarcoma cell lines from primary tumor and skip metastases. Acta Pharmacol Sin. 2008;29(3):325–32. 18298897 10.1111/j.1745-7254.2008.00756.x

[23] Berlin O, Samid D, Donthineni-Rao R, Akeson W, Amiel D, Woods VL Jr. Development of a novel spontaneous metastasis model of human osteosarcoma transplanted orthotopically into bone of athymic mice. Cancer Res. 1993;53(20):4890–5. 8402677

[24] Ritter J, Bielack SS. Osteosarcoma. Ann Oncol. 2010;21(Suppl 7):320–5. 10.1093/annonc/mdq276 20943636

[25] Zhao Z, Jia Q, Wu MS, Xie X, Wang Y, Song G, et al. Degalactotigonin, a natural compound from *Solanum nigrum* L., inhibits growth and metastasis of osteosarcoma through GSK3beta inactivation-mediated repression of the hedgehog/Gli1 pathway. Clin Cancer Res. 2018;24(1):130–44. 28951519 10.1158/1078-0432.CCR-17-0692

[26] Takebe N, Harris PJ, Warren RQ, Ivy SP. Targeting cancer stem cells by inhibiting Wnt, notch, and hedgehog pathways. Nat Rev Clin Oncol. 2011;8(2):97–106. 21151206 10.1038/nrclinonc.2010.196

[27] Pannuti A, Foreman K, Rizzo P, Osipo C, Golde T, Osborne B, et al. Targeting notch to target cancer stem cells. Clin Cancer Res. 2010;16(12):3141–52. 20530696 10.1158/1078-0432.CCR-09-2823 PMC3008160

[28] Liu W, Zhao Z, Wang Y, Li W, Su Q, Jia Q, et al. Dioscin inhibits stem-cell-like properties and tumor growth of osteosarcoma through Akt/GSK3/beta-catenin signaling pathway. Cell Death Dis. 2018;9(3):343. 29497056 10.1038/s41419-018-0363-x PMC5832770

[29] Luo X, Li L, Xu W, Cheng Y, Xie Z. HLY78 attenuates neuronal apoptosis via the LRP6/GSK3beta/beta-catenin signaling pathway after subarachnoid hemorrhage in rats. Neurosci Bull. 2020;36(10):1171–81. 32562163 10.1007/s12264-020-00532-4 PMC7532258

[30] Luo X, Li L, Zheng W, Gu L, Zhang X, Li Y, et al. HLY78 protects blood-brain barrier integrity through Wnt/beta-catenin signaling pathway following subarachnoid hemorrhage in rats. Brain Res Bull. 2020;162:107–14. 32565130 10.1016/j.brainresbull.2020.06.003

[31] Wang S, Yin J, Chen D, Nie F, Song X, Fei C, et al. Small-molecule modulation of Wnt signaling via modulating the Axin-LRP5/6 interaction. Nat Chem Biol. 2013;9(9):579–85. 23892894 10.1038/nchembio.1309

[32] Zhao SJ, Kong FQ, Jie J, Li Q, Liu H, Xu AD, et al. Macrophage MSR1 promotes BMSC osteogenic differentiation and M2-like polarization by activating PI3K/AKT/GSK3beta/beta-catenin pathway. Theranostics. 2020;10(1):17–35. 31903103 10.7150/thno.36930 PMC6929615

[33] Luo X, Zheng E, Wei L, Zeng H, Qin H, Zhang X, et al. The fatty acid receptor CD36 promotes HCC progression through activating Src/PI3K/AKT axis-dependent aerobic glycolysis. Cell Death Dis. 2021;12(4):328. 33771982 10.1038/s41419-021-03596-w PMC7997878

[34] Xu R, Song J, Ruze R, Chen Y, Yin X, Wang C, et al. SQLE promotes pancreatic cancer growth by attenuating ER stress and activating lipid rafts-regulated Src/PI3K/Akt signaling pathway. Cell Death Dis. 2023;14(8):497. 37542052 10.1038/s41419-023-05987-7 PMC10403582

[35] Brown HK, Tellez-Gabriel M, Heymann D. Cancer stem cells in osteosarcoma. Cancer Lett. 2017;386:189–95. 27894960 10.1016/j.canlet.2016.11.019

[36] Sluga M, Windhager R, Pfeiffer M, Ofner P, Lang S, Dominkus M, et al. Osteosarcoma and Ewing’s sarcoma–the most frequent malignant bone tumors in children–therapy and outcome. Z Orthop Ihre Grenzgeb. 2002;140(6):652–5. 12476389 10.1055/s-2002-36040

[37] Mirabello L, Troisi RJ, Savage SA. Osteosarcoma incidence and survival rates from 1973 to 2004: data from the surveillance, epidemiology, and end results program. Cancer. 2009;115(7):1531–43. 19197972 10.1002/cncr.24121 PMC2813207

[38] Escalante NK, von Rossum A, Lee M, Choy JC. CD155 on human vascular endothelial cells attenuates the acquisition of effector functions in CD8 T cells. Arterioscler Thromb Vasc Biol. 2011;31(5):1177–84. 21330602 10.1161/ATVBAHA.111.224162

[39] Georgiev H, Ravens I, Shibuya A, Forster R, Bernhardt G. CD155/CD226-interaction impacts on the generation of innate CD8(+) thymocytes by regulating iNKT-cell differentiation. Eur J Immunol. 2016;46(4):993–1003. 26689152 10.1002/eji.201546073

[40] Huang DW, Huang M, Lin XS, Huang Q. CD155 expression and its correlation with clinicopathologic characteristics, angiogenesis, and prognosis in human cholangiocarcinoma. Onco Targets Ther. 2017;10:3817–25. 28814880 10.2147/OTT.S141476 PMC5546808

[41] Yong H, Cheng R, Li X, Gao G, Jiang X, Cheng H, et al. CD155 expression and its prognostic value in postoperative patients with breast cancer. Biomed Pharmacother. 2019;115:108884. 31035013 10.1016/j.biopha.2019.108884

[42] Nishiwada S, Sho M, Yasuda S, Shimada K, Yamato I, Akahori T, et al. Clinical significance of CD155 expression in human pancreatic cancer. Anticancer Res. 2015;35(4):2287–97. 25862891

[43] O’Donnell JS, Madore J, Li XY, Smyth MJ. Tumor intrinsic and extrinsic immune functions of CD155. Semin Cancer Biol. 2020;65:189–96. 31883911 10.1016/j.semcancer.2019.11.013

[44] Zhang Q, Bi J, Zheng X, Chen Y, Wang H, Wu W, et al. Blockade of the checkpoint receptor TIGIT prevents NK cell exhaustion and elicits potent anti-tumor immunity. Nat Immunol. 2018;19(7):723–32. 29915296 10.1038/s41590-018-0132-0

[45] Luu HH, Kang Q, Park JK, Si W, Luo Q, Jiang W, et al. An orthotopic model of human osteosarcoma growth and spontaneous pulmonary metastasis. Clin Exp Metastasis. 2005;22(4):319–29. 16170668 10.1007/s10585-005-0365-9

[46] Zhang JX, Mai SJ, Huang XX, Wang FW, Liao YJ, Lin MC, et al. MiR-29c mediates epithelial-to-mesenchymal transition in human colorectal carcinoma metastasis via PTP4A and GNA13 regulation of beta-catenin signaling. Ann Oncol. 2014;25(11):2196–204. 25193986 10.1093/annonc/mdu439

[47] Adhikari AS, Agarwal N, Wood BM, Porretta C, Ruiz B, Pochampally RR, et al. CD117 and Stro-1 identify osteosarcoma tumor-initiating cells associated with metastasis and drug resistance. Cancer Res. 2010;70(11):4602–12. 20460510 10.1158/0008-5472.CAN-09-3463 PMC3139225

[48] Al-Hajj M, Clarke MF. Self-renewal and solid tumor stem cells. Oncogene. 2004;23(43):7274–82. 15378087 10.1038/sj.onc.1207947

[49] Reya T, Morrison SJ, Clarke MF, Weissman IL. Stem cells, cancer, and cancer stem cells. Nature. 2001;414(6859):105–11. 11689955 10.1038/35102167

[50] Yan GN, Lv YF, Guo QN. Advances in osteosarcoma stem cell research and opportunities for novel therapeutic targets. Cancer Lett. 2016;370(2):268–74. 26571463 10.1016/j.canlet.2015.11.003

[51] Tang QL, Zhao ZQ, Li JC, Liang Y, Yin JQ, Zou CY, et al. Salinomycin inhibits osteosarcoma by targeting its tumor stem cells. Cancer Lett. 2011;311(1):113–21. 21835542 10.1016/j.canlet.2011.07.016

[52] Zamanian M, Bazmandegan G, Sureda A, Sobarzo-Sanchez E, Yousefi-Manesh H, Shirooie S. The protective roles and molecular mechanisms of troxerutin (Vitamin P4) for the treatment of chronic diseases: a mechanistic review. Curr Neuropharmacol. 2021;19(1):97–110. 32386493 10.2174/1570159X18666200510020744 PMC7903491

