# Supplementary material for: Troxerutin suppresses the stemness of osteosarcoma via the CD155/SRC/β-catenin signaling axis
Source: Cell Mol Biol Lett. 2025 Apr 11;30:45. doi: 10.1186/s11658-025-00724-8 (PMC11992710; doi:10.1186/s11658-025-00724-8)
Supplement: Supplementary file 1 — Additional file 1. [file 11658_2025_724_MOESM1_ESM.docx]

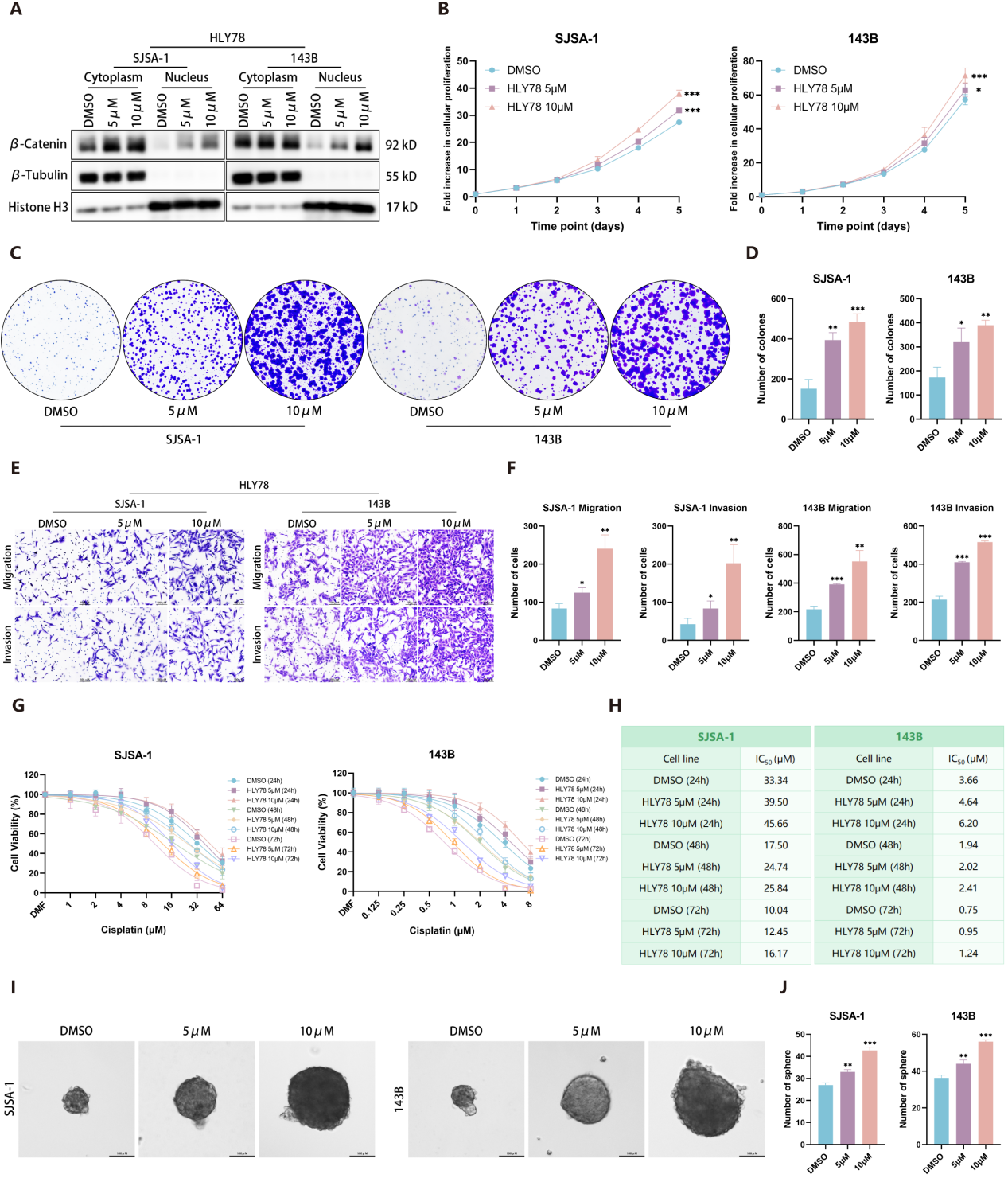


Figure S1. The malignant phenotype of OS cells exhibited an increase after being treated with HLY78. (A) The nuclear translocation of β-Catenin in JSA-1 and 143B cells following treatment with HLY78 was thoroughly assessed utilizing the Western blot. (B-D) The CCK-8 assay (B) and colony formation assay (C,D) were employed to assess the proliferative capacity of OS cells upon the addition of HLY78. (E,F) Utilizing the Transwell assay, the invasion and migration capacities of OS cells are evaluated. (G,H) The CCK-8 assay is employed to assess the resistance of OS cells towards cisplatin. (I) Representative images of the in vitro sphere formation assay. (J) The quantity of sphere in the in vitro sphere formation assay. **p*<0.05；***p*<0.01；****p*<0.001


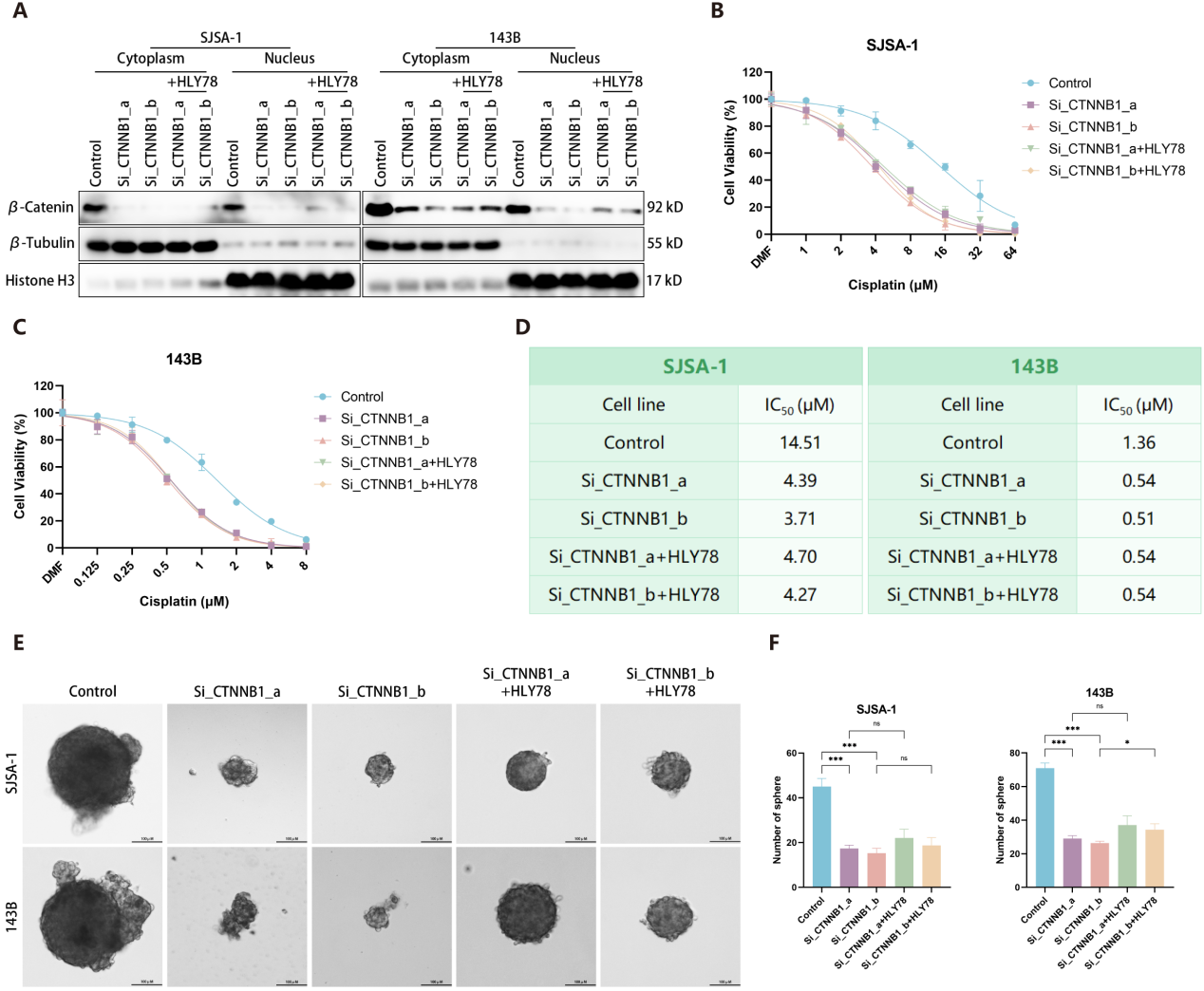


Figure S2. Upon the introduction of HLY78 into OS cells with downregulated CTNNB1, the restoration of their malignant phenotype was not particularly evident. (A) The nuclear translocation of β-Catenin in OS cells with knocked-down β-Catenin was evaluated using the western blot following the addition of HLY78. (B-D) The CCK-8 assay is employed to assess the resistance of OS cells towards cisplatin. (E) Representative images of the in vitro sphere formation assay. (F) The quantity of sphere in the in vitro sphere formation assay. *p<0.05；**p<0.01；***p<0.001


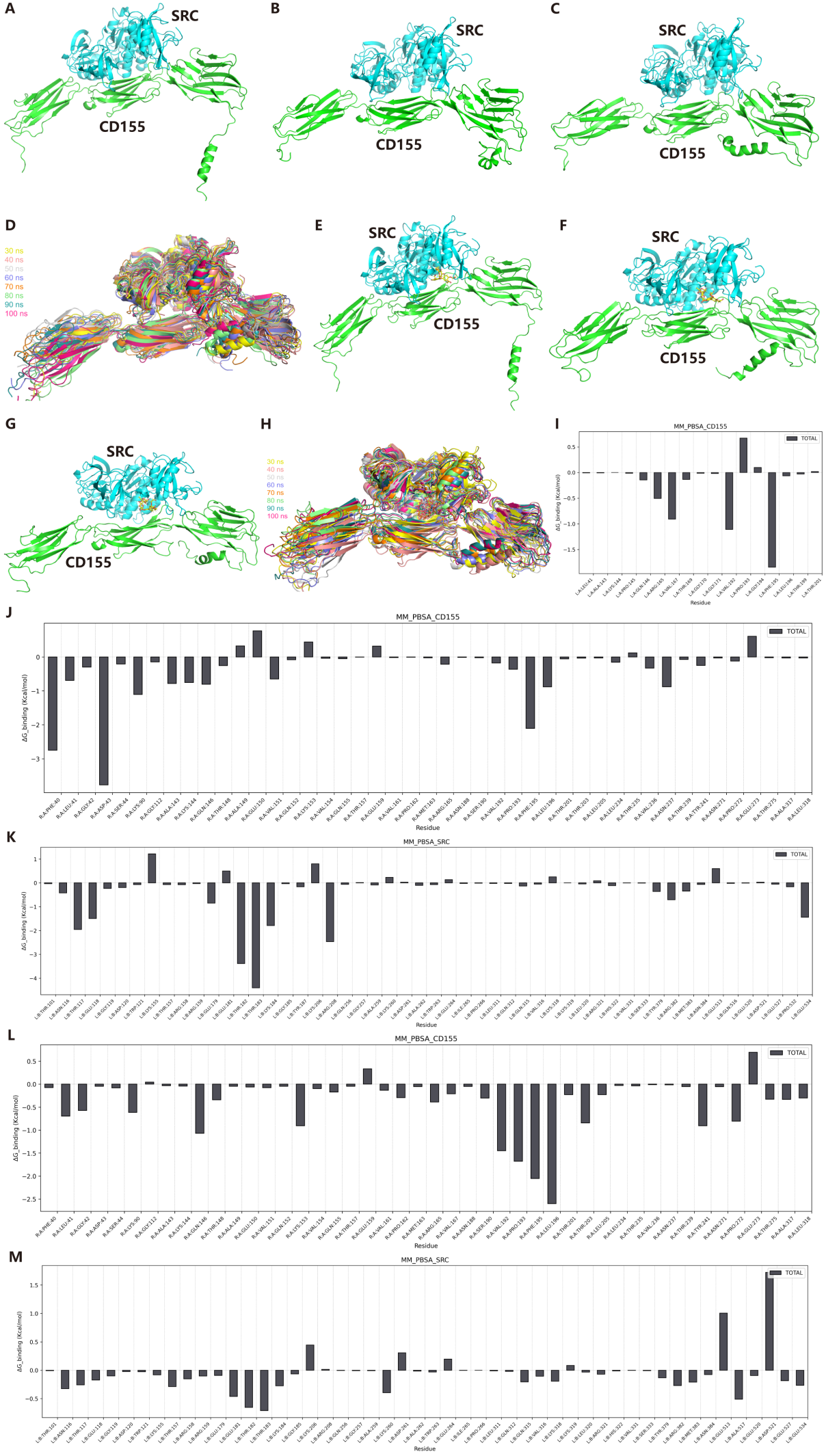


Figure S3. Molecular dynamics simulation of the structural changes between CD155 and SRC after the addition of Troxerutin, and the contribution of each residue to the change in binding free energy. (A) Conformation of the CD155-SRC complex at 0 ns in molecular dynamics simulations. (B) Conformation of the CD155-SRC complex at 10 ns in molecular dynamics simulations. (C) Conformation of the CD155-SRC complex at 20 ns in molecular dynamics simulations. (D) Conformation of the CD155-SRC complex at 30-100 ns in molecular dynamics simulations. (E) Conformation of the CD155-SRC complex at 0 ns during molecular dynamics simulations with Troxerutin added. (F) Conformation of the CD155-SRC complex at 10 ns during molecular dynamics simulations with Troxerutin added. (G) Conformation of the CD155-SRC complex at 20 ns during molecular dynamics simulations with Troxerutin added. (H) Conformation of the CD155-SRC complex at 30-100 ns during molecular dynamics simulations with Troxerutin added. (I) Decomposition of free energy residues in the binding of CD155 and Troxerutin. (J,K) Residue decomposition of the binding free energy between CD155 (J) and SRC (K). (L,M) Residue decomposition of binding free energy between CD155 (L) and SRC (M) after adding Troxerutin.
